# Influence of Tooth Whitening After Resin Infiltration on the Shear Bond Strength of Orthodontic Brackets

**DOI:** 10.3290/j.jad.c_2772

**Published:** 2026-08-05

**Authors:** Christoph Matthias Schoppmeier, Li Sun, Malin Janson, Isabelle Graf, Bert Braumann, Max von Kohout, Anna Greta Barbe, Michael Jochen Wicht

**Affiliations:** a Christoph Matthias Schoppmeier Dentist and Senior Physician, University of Cologne, Faculty of Medicine and University Hospital Cologne, Polyclinic for Operative Dentistry and Periodontology, Cologne, Germany. Writing – review and editing, writing – original draft, visualization, resources, project administration, methodology, investigation, formal analysis, data curation, conceptualization.; b Li Sun Dentist, University of Cologne, Faculty of Medicine and University Hospital Cologne, Polyclinic for Operative Dentistry and Periodontology, Cologne, Germany. Writing – review and editing, methodology, data curation.; c Malin Janson Dentist, University of Cologne, Faculty of Medicine and University Hospital Cologne, Department of Prosthetic Dentistry, Cologne, Germany. Writing – review and editing, writing – original draft, methodology, data curation.; d Isabelle Graf Orthodontist, Senior Physician, University of Cologne, Faculty of Medicine and University Hospital Cologne, Department of Orthodontics and Dentofacial Orthopedics, Cologne, Germany. Writing – review and editing, writing – original draft, methodology.; e Bert Braumann Orthodontist, Head of Department, University of Cologne, Faculty of Medicine and University Hospital Cologne, Department of Orthodontics and Dentofacial Orthopedics, Cologne, Germany. Writing – review and editing, validation, supervision, methodology.; f Max von Kohout Statistician, Faculty of Medicine and University Hospital Cologne, Institute of Medical Statistics and Computational Biology, University of Cologne, Cologne, Germany. Data curation, formal analysis, visualization.; g Anna Greta Barbe Dentist, Head of Department, University of Cologne, Faculty of Medicine and University Hospital Cologne, Polyclinic for Operative Dentistry and Periodontology, Cologne, Germany. Supervision, writing – review and editing, conceptualization, project administration.; h Michael Jochen Wicht Dentist, Senior Physician, University of Cologne, Faculty of Medicine and University Hospital Cologne, Polyclinic for Operative Dentistry and Periodontology, Cologne, Germany. Writing – review and editing, writing – original draft, validation, methodology, project administration.

**Keywords:** bleaching, bracket, resin infiltration, shear bond strength

## Abstract

**Purpose:**

To determine the influence of different bleaching methods on the shear bond strength (SBS) of orthodontic brackets bonded to resin-infiltrated (Icon, DMG) extracted human enamel.

**Methods and Materials:**

64 extracted wisdom teeth were randomized into four groups (n = 16): 1. (–)control (no treatment), 2. (+)control (RI), 3. RI followed by 25% H_2_O_2_ (IOB) and 4. RI followed by 10% carbamide peroxide bleaching (HB). After a standardized demineralization protocol, RI and bleaching were performed according to the manufacturer’s instructions prior to storage for 14 days. Brackets (Mini Diamond Twin, Ormco) were bonded buccally and lingually (Transbond XT, 3M). SBS values (MPa, traverse speed 0.5 mm/min, Zwick Roell) and the Adhesive Remnant Index (ARI) were determined on the buccal side. Following 5000 thermal cycles (5–55°C; RC 20 CS Lauda), SBS and ARI values for the lingual brackets were determined analogously. Data were analyzed with mixed ANOVA and Kruskal–Wallis and Wilcoxon tests at a 0.05 significance level.

**Results:**

Differences in SBS were observed among groups (F(3, 64) = 8.01, P < 0.001). HB exhibited the lowest SBS values (14.99 +/- 6.42 MPa; 10.84 +/- 3.61 MPa), differing significantly from the negative control (P = 0.041; P = 0.002 after thermocycling). No significant differences were noted between IOB and HB. Thermocycling reduced SBS values across all groups (F(3, 64) = 25.83, P < 0.001).

**Conclusion:**

RI followed by bleaching negatively impacts bracket bonding strength, with a significant reduction observed after home bleaching, while in-office bleaching showed no such effect.

Children and adolescents are at a higher risk of developing white spot lesions (WSL) compared to adults. WSLs might appear as a consequence of impaired oral hygiene during orthodontic treatment, with a prevalence of up to 50%.^25^ Interestingly, between 11% and 24% of all patients exhibit WSLs before orthodontic treatment,^45^ indicating that a significant proportion of this patient group receives orthodontic treatment despite the presence of initial WSLs. In the wake of social change and the increasing use of social media, adolescents and especially young adults are nowadays more sensitive about their appearance.^8^ Whitish opacities (including WSL), particularly in the esthetically relevant area of the anterior teeth, can evoke negative associations and cause a feeling of unattractiveness.^1,20^ Accordingly, the desire for esthetic rehabilitation arises in a period that often clashes with the timing of orthodontic treatment.^22^ Over time, resin infiltration (RI) (Icon, DMG, Hamburg, Germany) has established itself as a microinvasive treatment option for masking and stabilizing WSLs.^23^ The primary component of the resin infiltrant, triethylene glycol dimethacrylate (TEGDMA), enables the infiltrant to penetrate deeply into the lesion body through capillary forces, thereby sealing it and protecting the infiltrated enamel from further demineralization.^19^ Since air and water inclusions within the lesion body are sealed by the infiltrant and the latter has a similar optical refractive index to healthy enamel, the white opacities are masked at the same time.^47^ However, the low-viscosity resin infiltrant shows the potential for higher water absorption over time, leading to hygroscopic expansion and hydrolytic degradation of the infiltrant.^29,38^ The increased water absorption and rougher surface texture of infiltrated teeth compared to sound enamel result in a greater potential for discoloration.^2,31,41^ As the infiltrant itself has a yellowish color, a significant proportion of patients seek to improve the appearance of their teeth over time through professional bleaching.^9^

In-office bleaching is an established procedure for treating WSLs and often uses hydrogen peroxide (HP) or carbamide peroxide (CP).^16,40^ The bleaching process can be performed either as in-office bleaching (IOB) in a single session with a high concentration of HP and optional light activation or as home bleaching (HB), where patients use custom-fitted trays and bleaching agents with lower concentrations of CP.^34,40^ This method allows for gradual whitening over an extended period. Both procedures are frequently combined with resin infiltration to mask discolorations by increasing enamel brightness and reducing yellowing.^40,48^ Studies have shown that resin infiltration prior to bleaching can mitigate the negative effects, such as the reduction in the hardness of WSL enamel.^13^ Additionally, it has been demonstrated that pre-bleaching in cases of fluorotic defects leads to significantly better esthetic outcomes, as it helps to reduce the intrinsic discoloration and improve the overall brightness of the enamel surface. This process enhances the subsequent penetration of resin infiltrants by creating a more uniform color baseline, thereby optimizing the final esthetic results and ensuring a more natural appearance.^40^ Furthermore, resin infiltration after bleaching has proven effective in removing surface stains and reducing the yellowing of the infiltrant, resulting in better bleaching results and higher patient satisfaction.^49^ Once the teeth have been bleached, patients often exhibit a heightened and immediate interest in orthodontic treatment.^10^ Therefore, bonding of brackets to infiltrated and already bleached enamel surfaces might become a frequent procedure during orthodontic routine care.^21^ The establishment of a reliable adhesive bond between the bracket and enamel is essential for the success of the planned orthodontic treatment.^21^ Costenoble et al suggested that bracket bonding should ideally be performed shortly after RI.^14^ Additionally, numerous studies have reported similar bond strengths between sound enamel and resin-infiltrated enamel, indicating that RI has minimal to no negative impact on adhesive performance.^14,36,46^ However, studies concerning the effects of bleaching have demonstrated that the shear bond strength (SBS) of bonded brackets is reduced immediately following bleaching. This reduction may be attributed to changes in the enamel surface roughness, structural alterations due to the loss of prism structure, modifications in organic material, calcium loss, and a decrease in microhardness.^12,44^ Furthermore, residual oxygen released from the bleaching agent can inhibit resin infiltration into the bleached enamel or impede resin polymerization. The application of antioxidants can neutralize the oxygen radicals within the enamel structures immediately post-bleaching, thereby allowing for the immediate bonding of brackets.^5^ Alternatively, a waiting period of 14 days can be observed to ensure the radicals are adequately washed out.^12,40^

However, the question arises as to whether bleaching of infiltrated teeth can be effectively performed prior to orthodontic treatment, as will probably be the case more often in the future with young people who also use bleaching before orthodontic treatment. At present, while the effect of home and in-office bleaching on the color stability of resin-infiltrated white-spot lesions has recently been quantified,42 comprehensive data ^o^n the impact of bleaching on resin-infiltrated enamel prior to orthodontic bonding are still lacking. Additionally, since orthodontic adhesives undergo thermal variations in the oral cavity, it is essential to determine whether such temperature variations induce stress on the adhesive interface, influencing bond strength.^12,17^ Thus, this *in vitro* study evaluated the influence of different bleaching methods on the shear bond strength of orthodontic brackets bonded to resin-infiltrated, extracted human enamel.

The null hypotheses for this study are:

1. Bleaching does not reduce the shear bond strength of orthodontic brackets bonded to resin-infiltrated enamel.
2. There is no difference in the shear bond strength of orthodontic brackets between the two bleaching procedures: in-office bleaching (IOB) and home bleaching (HB).

## METHODS AND MATERIALS

Approval for the *in vitro* study was obtained from the local board of ethics (23-1472, approval date 28-03-2024). The sample size was determined using G*Power software (Version 3.1.9.6, Franz Faul, University of Kiel, Germany). With a 1:1 ratio between the comparison groups, a total sample of 64 wisdom teeth was required to achieve a statistical power of 80%, based on an effect size (Cohen’s d) of 0.659 and a significance level of 5% (α = 0.05).

Sixty-four caries-free wisdom teeth (equally distributed between upper and lower jaws) were extracted for reasons unrelated to the objectives of this study with informed consent and stored at 8°C in distilled water with 0.1% thymol to inhibit bacterial growth and prevent dehydration. Exclusion criteria encompassed teeth with caries, attrition, fractures, cracks, restorations, surface anomalies, and any pretreatment with hydrogen peroxide, formalin, or alcohol. Each sample was examined at 50× magnification to confirm the integrity of the enamel. Soft tissue remnants and calculi were removed using periodontal curettes (413/414 Universal Curette, Hu-Friedy, Chicago, IL, USA). Each tooth was positioned vertically and embedded up to two-thirds of its root in chemically activated acrylic resin (Palapress vario transparent, Kulzer, Hanau, Germany). Subsequently, the crowns were cleaned with a fluoride-free pumice and a rubber cup at low speed to ensure removal of all residual debris. After cleaning, the teeth were rinsed with water. The teeth were randomly assigned to four groups (n = 16).

Group 1 – (–) negative control: Sound enamel
Group 2 – (+) control: Demineralization + RI
Group 3 – IOB (in-office bleaching): Demineralization + RI + 25% H_2_O_2_
Group 4 – HB (home bleaching): Demineralization + RI + 10% CP

**Fig 1:**
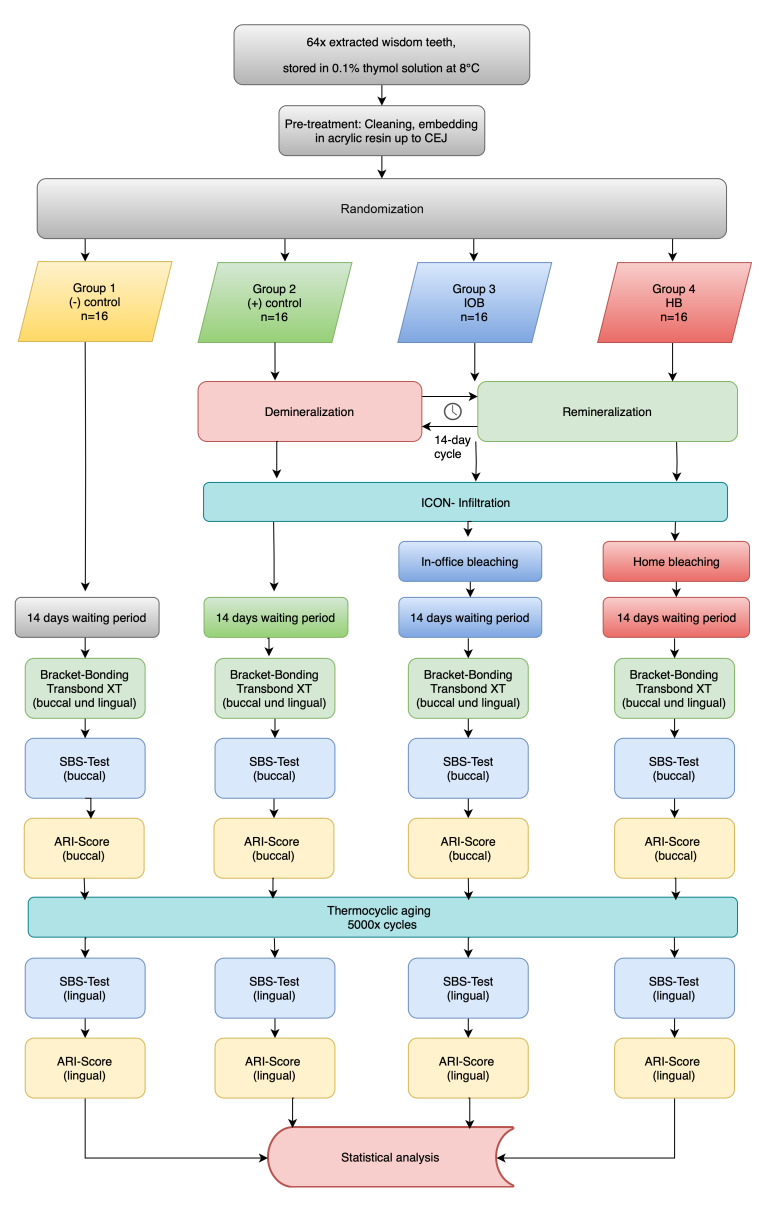
Study flowchart.

The specific treatment measures for each group can be found in Figure 1. Details regarding the brand names, manufacturers, batch numbers, chemical compositions, and applications of the materials used in this study are provided in Table 1.

**Table 1:** Materials, manufacturers, and lot numbers of the products used in this study

Material	Manufacturer	Composition	Lot No.
Icon – Etch	DMG (Hamburg, Germany)	15% hydrochloric acid	261396
Icon – Dry	DMG (Hamburg, Germany)	99% ethanol	261396
Icon – Infiltrant	DMG (Hamburg, Germany)	TEGDMA, initiators, additives	261396
ZOOM!	Discus Dental Europe (Rotterdam, Netherlands)	25% hydrogen peroxide, potassium hydroxide, eugenol, 2,6-di-tertiary-butyl-4-methylphenol	23279010
Opalescence PF 10%	Ultradent Products (UT, USA)	3.5% hydrogen peroxide equivalent Glycerin, Water, urea peroxide, Xylitol, Carbomer, PEG-6, Sodium Hydroxide, EDTA, potassium nitrate, 0.11% fluoride ion	BXJ83
Etchant Gel	Ultradent Products (UT, USA)	35% phosphoric acid water, cobalt aluminate blue spinel, glycol, siloxane	BTF9K
Transbond XT	3M Unitek (Monrovia, CA, USA)	Transbond XT Primer: Bis-GMA, TEGDMA, 4-dimethylaminobenzene ethanol, camphorquinone, hydroquinone Transbond XT Adhesive: Bis-GMA, Bis-EMA, acrylate, monomers, filler	PL8SD
Mini Diamond Twin Bracket	Ormco (CA, USA)	Stainless steel	10239290N
Abbreviations: Bis-EMA, Ethoxylated bisphenol-A dimethacrylate; Bis-GMA, bisphenol A-glycidyl dimethacrylate; TEGDMA, Triethyleneglycol dimethacrylate			

### Demineralization

For Groups 2–4, tooth crowns were exposed to a cariogenic environment to induce the formation of WSL. Details on the composition of the demineralizing and remineralizing solutions, as well as the artificial saliva, can be found in Appendix 1. Each sample underwent a 16-h demineralization period followed by a rinse with deionized water and a subsequent 8-h remineralization, completing the 24-h cycle according to Hu et al.^28^ This process was repeated daily for 14 days, with both neutral and acidic solutions being refreshed daily. After the demineralization cycle, all samples (including Group 1) were maintained in artificial saliva at 37°C for 56 h.^24^ Presence of WSLs was confirmed using Diagnodent (KaVo Dental, Biberach, Germany) with a threshold value of 13.^38^

### Icon

In Groups 2–4, WSLs were treated with Icon (DMG, Hamburg, Germany) as per the manufacturer’s protocols.^4^ Treatment steps included a hydrochloric acid etch (15% HCl, Icon Etch, DMG, Hamburg, Germany) for 2 min, followed by a 30-s rinse and a 30-s drying with ethanol (Icon Dry, DMG, Hamburg, Germany). A dual application of a low-viscosity resin infiltrant (Icon Infiltrant, DMG, Hamburg, Germany) was performed, first for 3 min and then for 1 min. Each application was followed by a 40-s light-curing (1200 mW/cm^2^, Bluephase Style, Ivoclar Vivadent, Schaan, Liechtenstein), maintaining a 2-mm distance from the surface.

### Bleaching

In Groups 3 and 4, different bleaching treatments were implemented. Group 3 (IOB) underwent a simulated in-office bleaching with 25% hydrogen peroxide (ZOOM!; Discus Dental Europe, Rotterdam, Netherlands), consisting of four 15-min cycles following the manufacturer’s instructions. Group 4 (HB) received a regimen in accordance with a home bleaching protocol using 10% carbamide peroxide (Opalescence PF 10%, Ultradent Products, South Jordan, UT, USA) applied to buccal and lingual surfaces 8 h for 5 days, according to the manufacturer’s guidelines. Studies indicate comparable whitening effects between in-office and at-home treatments.^15^ Samples were then stored in distilled water for 14 days. Bracket bonding for all groups was conducted 2 weeks after bleaching to ensure the complete removal of oxygen radicals, similar to a clinical setting.

### Bracket Bonding

All samples underwent etching with 35% phosphoric acid gel for 30 s, were rinsed with water, and then air-dried. Two stainless-steel brackets (Mini Mesh Twin Upper Bicuspid Universal 0.22 slot, 10.65 mm^2^ of area, Ormco, USA) were bonded to both the buccal and lingual surfaces of each tooth using Transbond XT adhesive (3M Unitek, Monrovia, CA, USA), as per the manufacturer’s instructions, by an experienced orthodontist who was blinded regarding group assignment. Excess adhesive was meticulously removed using microbrushes. The brackets were light-cured (1200 mW/cm^2^, Bluephase Style, Ivoclar Vivadent, Schaan, Liechtenstein) from cervical, incisal, mesial, and distal directions for 15 s each. All bonded samples were stored in distilled water at 37°C for 24 h.^21^

### SBS

Shear bond strength (SBS) testing was conducted using a Zwick universal testing machine (zwickiLine Z0.5 TN, Zwick Roell, Ulm, Germany). Samples were fixed in a custom-designed holder to ensure consistent application of force and direction of the debonding force. A chisel-shaped stainless-steel rod was employed for the test, with a crosshead speed set at 0.5 mm/min. The force was applied parallel to the enamel surface in an occluso-gingival direction at the interface between the bracket base and enamel. The load at failure was recorded in Newtons and converted to megapascals (MPa) to calculate the shear bond strength using the formula S = F/A, where S represents the shear strength, F is the load at failure (N), and A denotes the adhesive area (mm^2^). Initially, only the buccal brackets were tested.

### ARI Failure Mode

The Adhesive Remnant Index (ARI) and the location of failure were evaluated immediately after each shear bond strength test, utilizing a microscope (VHX-5000, Keyence, Osaka, Japan) at 50× magnification. The ARI was evaluated based on the 4-point scale developed by Årtun and Bergland^3^: 0 indicates no adhesive remaining on the tooth; 1 signifies less than half of the adhesive remains on the tooth; 2 denotes more than half of the adhesive remains on the tooth; and 3 means all of the adhesive remains on the tooth. The ARI scoring was performed by an independent examiner who was blinded to the experimental conditions.

### Thermocycling Aging

For the aging process, samples underwent 5,000 thermocycling cycles between 5°C and 55°C, with a 30-s dwell time and a 5-s transfer time using an RC 20 CS Lauda machine (Lauda-Königshofen, Germany). Subsequently, SBS tests and ARI assessments were conducted on the lingual surfaces.

### Scanning Electron Microscopy (SEM)

To analyze the adhesive interface, one random sample from each group was prepared according to the method of Costenoble et al.^14^ Samples were dried and coated with a gold-palladium layer using a sputter coater (Q150T Plus, Quorum, East Sussex, UK) to enhance electron conductivity. Surface morphology was examined using a Prisma E SEM (Thermo Fisher Scientific, Waltham, MA, USA), capturing images at magnifications ranging from 500× to 4,000× (1536 × 1024 pixels).

### Statistical

Statistical analyses were conducted using IBM SPSS Statistics 24.0 (IBM Corp., Armonk, NY, USA). Descriptive statistics, including mean, standard deviation, minimum, and maximum values for shear bond strength, were calculated for each group. The Kolmogorov–Smirnov test was used to assess the normality of the data. Homogeneity of variances across the groups was verified using Levene’s test. Subsequent analyses involved parametric tests; a mixed ANOVA was used to evaluate differences in shear bond strengths among the groups, followed by a Bonferroni post-hoc test for multiple comparisons. For the ARI scores, non-parametric Kruskal–Wallis and Wilcoxon tests were used, and significant differences between groups were determined. Furthermore, a Spearman correlation was conducted to investigate the relationship between SBS and ARI scores. A *P* value of less than 0.05 was considered statistically significant for all tests.

## RESULTS

**Fig 2:**
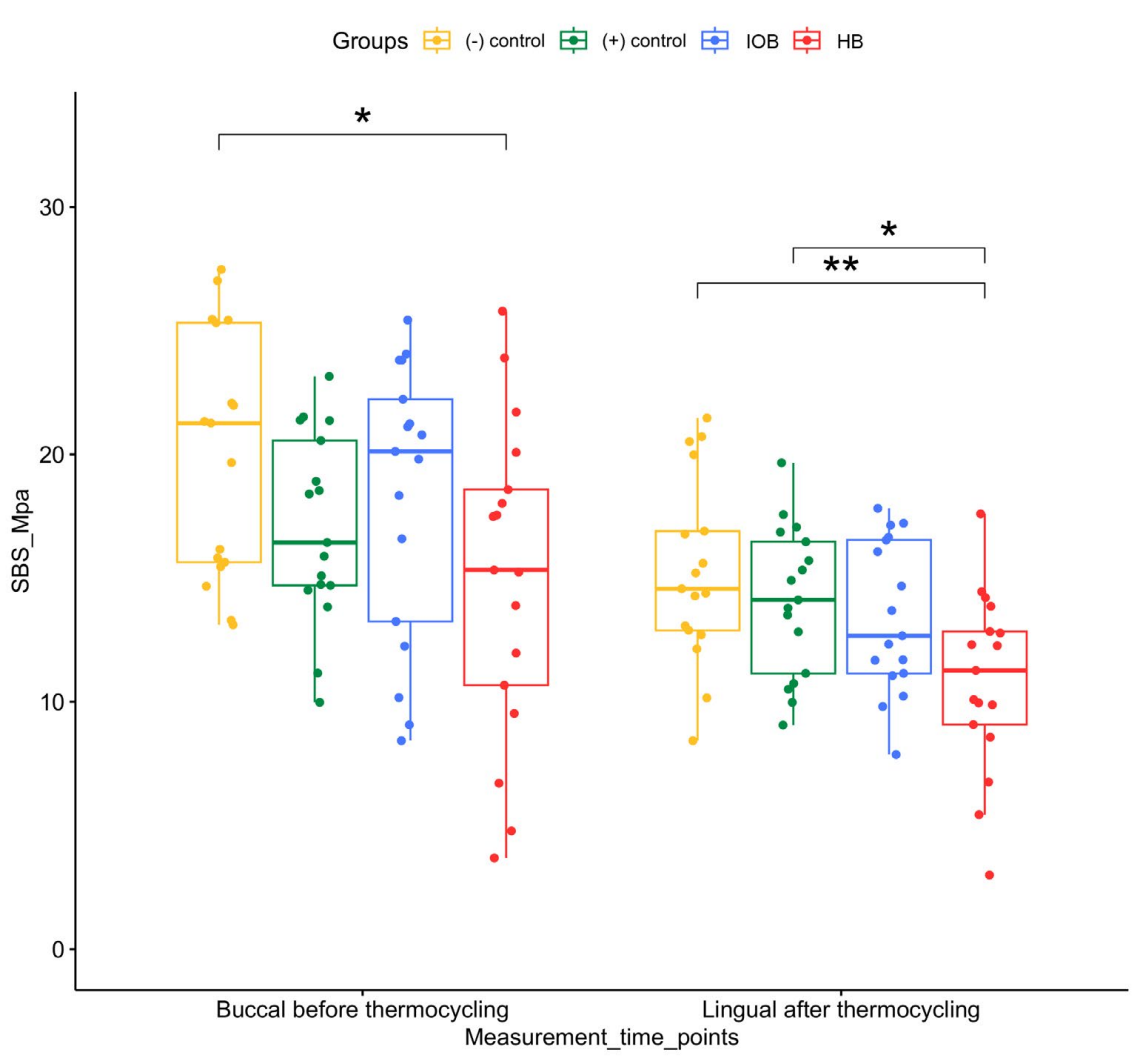
Box plots of shear bond strength (MPa) for the treatment groups before and after thermocycling aging; boxes show the median and interquartile range, whiskers the 1.5xIQR, dots the individual measurements. Groups marked with * are significantly different at *P *< 0.05, and those marked with ** are highly significant at *P* < 0.01. Abbreviation: MPa, Megapascal.

General assumptions of the conducted mixed ANOVA model were confirmed (*P* > 0.05) at both time points. Thermocycling significantly reduced SBS values in all groups (F(3, 64) = 25.83, *P* < 0.001 η2= 0.17). Significant results were also obtained for the group effect (F(3, 64) = 8.01, *P* < 0.001, η2= 0.11). SBS means and standard deviations are presented in Figure 2. Before and after thermocycling, group 1 (–) control showed the highest SBS values (20.07 ± 4.98 MPa; 15.28 ± 3.75 MPa). Group 4 (HB) demonstrated the lowest shear bond strength (14.99 ± 6.42 MPa; 10.84 ± 3.61 MPa), consistently showing a significant difference from group 1 at all time points (*P* = 0.041; *P* = 0.002 after thermocycling). Additionally, a significant difference was observed between group 4 and group 2 after thermocycling (*P* = 0.042). No significant differences were found between the bleaching procedures; the SBS values of group 3 (IOB) were similar to those of group 2.

Table 2 displays the frequency distribution of ARI scores after debonding. Significant differences in ARI scores were observed between groups both before and after thermocycling. Specifically, significant differences were found between the control group and the HB group before thermocycling (*P* = 0.042), which became more pronounced after thermocycling (*P* = 0.011). The Wilcoxon test revealed a significant reduction in ARI scores from before to after thermocycling (*P* < 0.001).

**Table 2:** ARI scores n (%) for all groups before and after thermocycling. Between-group comparisons were tested with the Kruskal-Wallis test

Group	ARI 0	ARI 1	ARI 2	ARI 3	*P*
before thermocycling (buccal)					
Group 1 – control (sound enamel)	0 (0)	4 (25.0)	6 (37.5)	6 (37.5)	*0.042*
Group 2 + control (demin. + RI)	2 (12.5)	5 (31.3)	3 (18.8)	6 (37.5)	
Group 3 IOB (demin. + RI + 25% H_2_O_2_)	1 (6.3)	5 (31.3)	5 (31.3)	5 (31.3)	
Group 4 HB (demin. + RI + 10% CP)	5 (31.3)	5 (31.3)	4 (25.0)	2 (12.5)	
after thermocycling (lingual)					
Group 1 – control (sound enamel)	1 (6.3)	6 (37.5)	6 (37.5)	3 (18.8)	*0.011*
Group 2 + control (demin. + RI)	3 (18.8)	5 (31.3)	7 (43.8)	1 (6.3)	
Group 3 IOB (demin. + RI + 25% H_2_O_2_)	4 (25.0)	7 (43.8)	4 (25.0)	1 (6.3)	
Group 4 HB (demin. + RI + 10% CP)	6 (37.5)	8 (50.0)	2 (12.5)	0 (0)	
Abbreviations: ARI, Adhesive Remnant Index; HB, Home bleaching; IOB, In-office bleaching; Demin., Demineralization; RI, Resin infiltration					

Additionally, the relationship between ARI scores and SBS was assessed using Spearman correlation coefficients. A strong, positive correlation was observed before thermocycling (Spearman’s rho = 0.81, *P* < 0.001), which remained significant after thermocycling (Spearman’s rho = 0.78, *P* < 0.001).

**Fig 3a and b:**
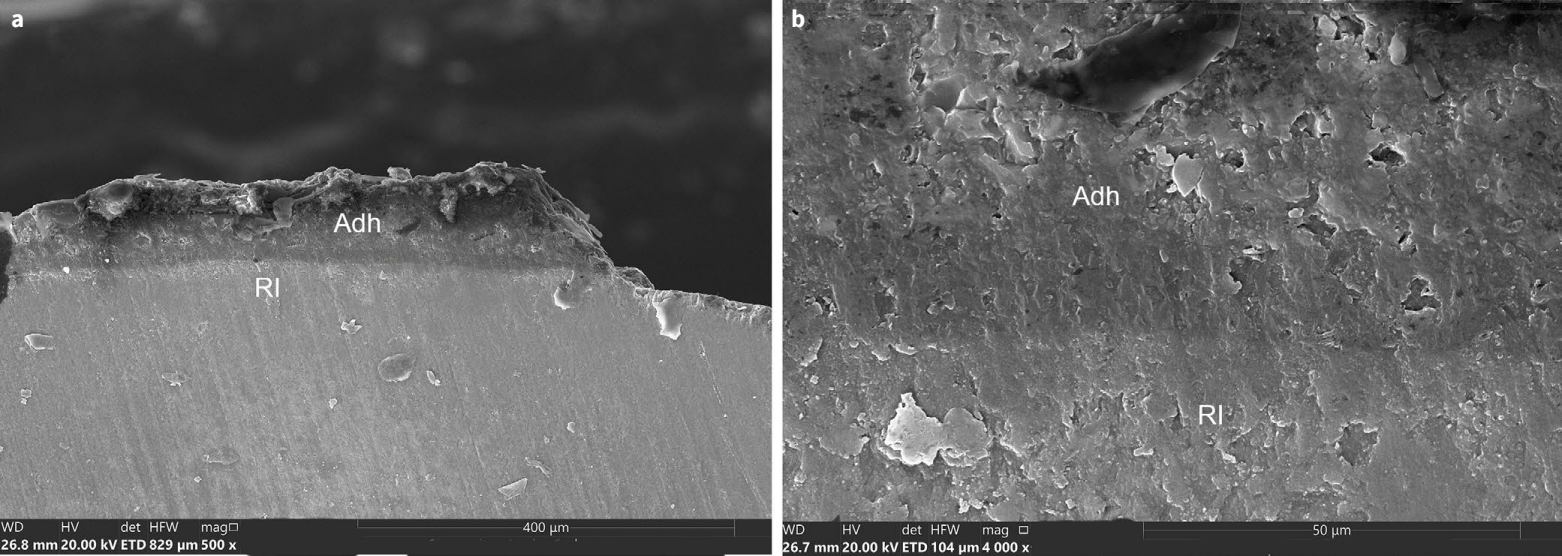
Micrograph of the (–) control showing uniform and regular resin tags of consistent length and close contact between the adhesive (Adh) and sound enamel at two different magnifications: (a) 500×, (b) 4000×.

**Fig 4a and b:**
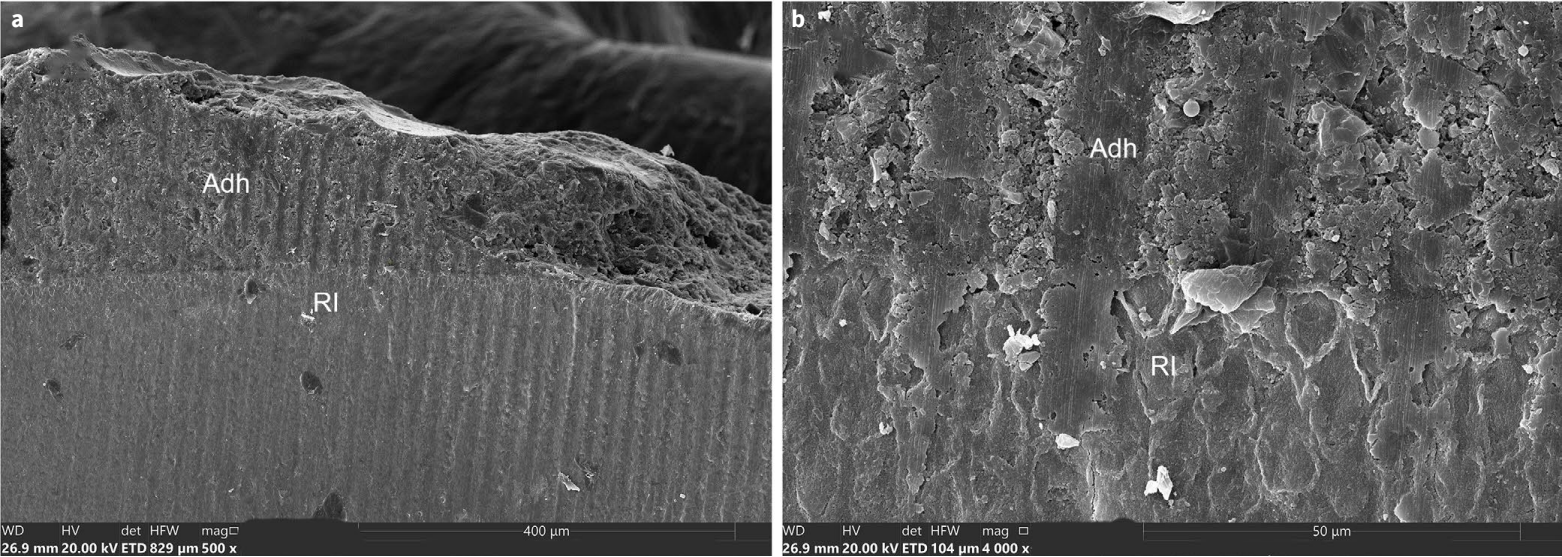
Micrograph of (+) control. Similar close contact between enamel, RI, and adhesive (Adh) at two different magnifications: (a) 500×, (b) 4000×.

**Fig 5a and b:**
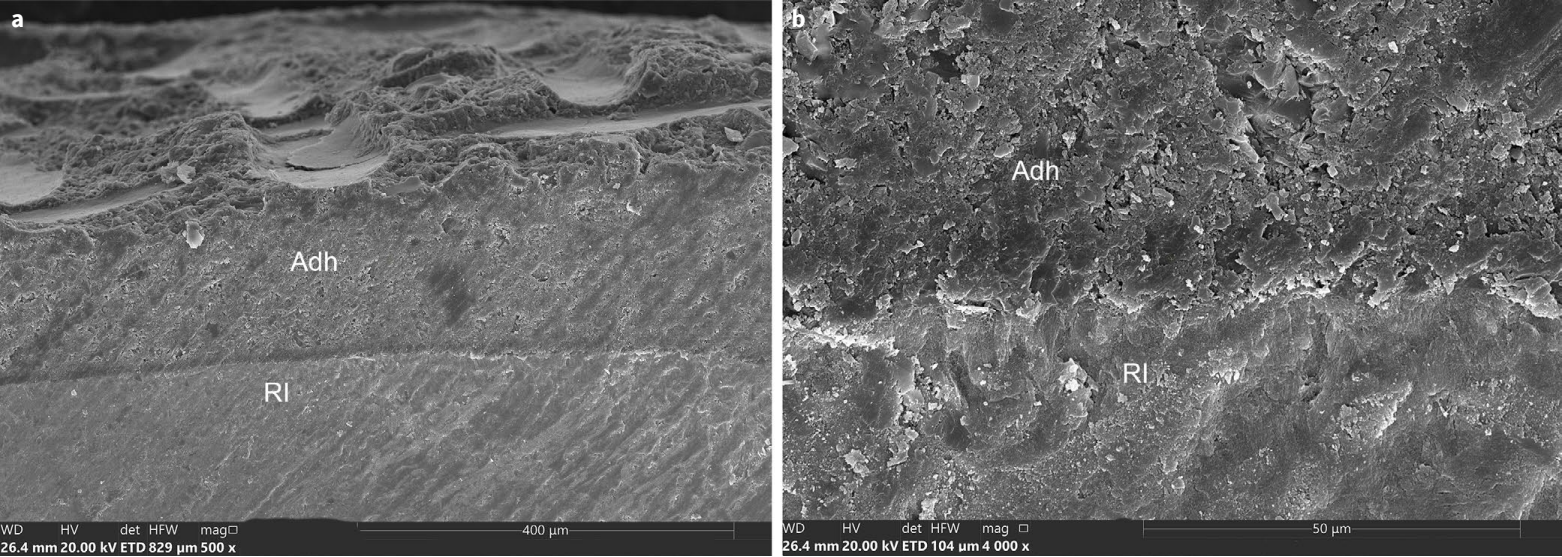
Micrograph of IOB with small voids or increased roughness between the RI layer and adhesive (Adh) at two different magnifications: (a) 500×, (b) 4000×.

**Fig 6a and b:**
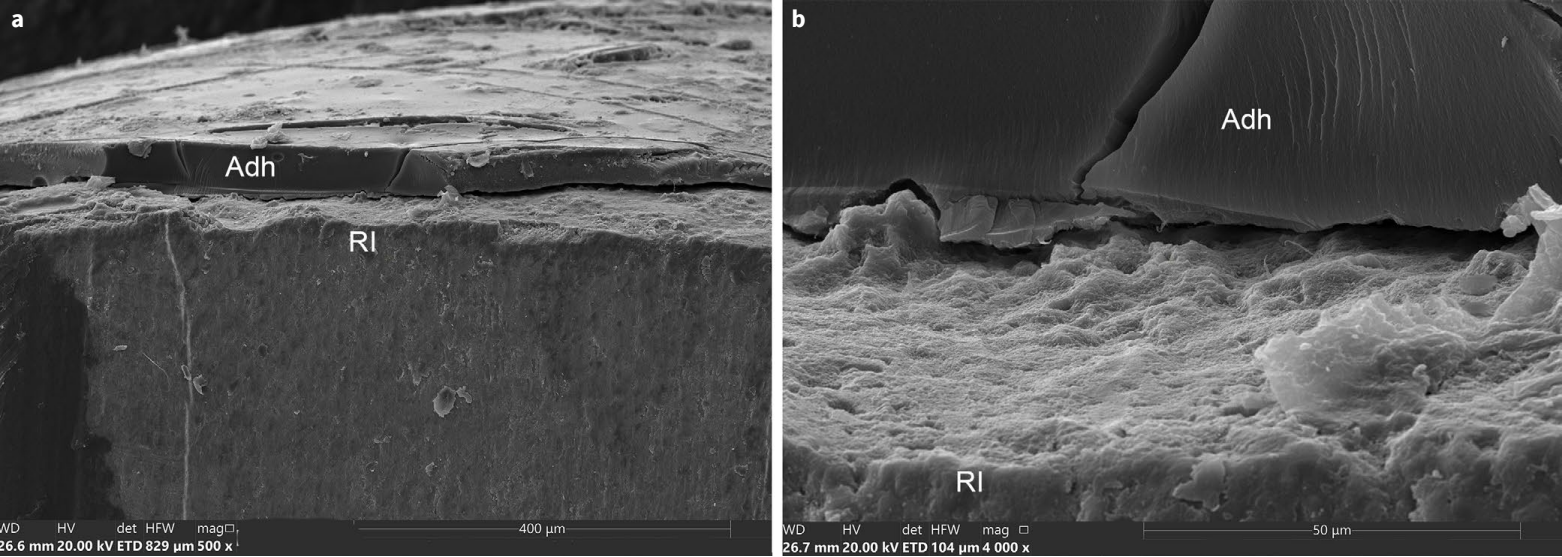
Micrograph of HB. The image shows the absence of the hybrid layer between RI and adhesive (Adh), accompanied by gap formation at two different magnifications: (a) 500×, (b) 4000×.

Figures 3 to 6 illustrate SEM images of the examined groups at 500× magnification (“a”) and 4000× magnification (“b”). Group 1 exhibited uniform and regular resin tags (RI) with consistent penetration depth. It was observed that there was a close contact between the resin tags and the adhesive. The SEM images of Groups 1 to 3 (Figs 3 to 5) also demonstrated a homogeneous hybrid layer with consistent infiltration penetration, which evenly covered the tooth surface and was well co-polymerized with the adhesive. However, in the IOB group, several voids and small gaps between the adhesive and RI were observed at the transition zone. Group 4 (HB) exhibited a retentive etching pattern in the enamel, an inhomogeneous and partially lost RI layer, and significant gap formation with the adhesive (Fig 6).

## DISCUSSION

In this study, the bond strength of orthodontic brackets to demineralized, resin-infiltrated, and bleached enamel was determined before and after thermocycling of the bonded teeth. The first hypothesis was partially rejected, as treating WSLs with RI followed by home bleaching with 10% carbamide peroxide significantly reduced the bonding efficacy, while bleaching with 25% hydrogen peroxide did not. The second hypothesis could not be rejected, as no differences were found in bonding efficacy between the two bleaching methods. Additionally, thermocycling markedly reduced shear bond strengths across all treatment groups.

Studies have shown that the bond strength of composite resins to enamel decreases following a bleaching procedure, both at home and in the dental office.^12,18^ The use of bleaching agents negatively affects the penetration depth of bonding, primarily due to the loss of enamel prisms.^33^ Additionally, it increases the roughness and porosity of the enamel and reduces its microhardness.^27^ Free oxygen radicals from the bleaching agents penetrate the enamel-resin interface, obstruct the resin penetration, and disrupt resin polymerization.^40^ Similar observations were made regarding bonding on previously bleached enamel, leading to the recommendation that bracket bonding should be performed after 14 days.^7,17^ Recent data indicate that the average SBS values on sound enamel were lower in home-bleaching groups.^11^ During the bleaching process, 10% carbamide peroxide dissociates into 3.5% hydrogen peroxide and urea, with the hydrogen peroxide subsequently breaking down into oxygen radicals and water. The concentration of these radicals in the tooth varies depending on the duration of contact with the bleaching gel, which explains the lower SBS values when using carbamide peroxide compared to hydrogen peroxide.^26^ In-office bleaching with 25% hydrogen peroxide was applied four times for 15 min each, while carbamide peroxide was used for 5 days, 8 h per day. Consequently, the concentration of these radicals within the tooth can vary depending on the contact duration with the bleaching gel, leading to a time-dependent reduction in bond strength.^11,18^ Artificial carious lesions were created using the methods of previous studies and exhibited the characteristic histological structures, including a pseudo-intact surface layer.^21^ These artificially induced lesions offer the advantage of increased homogeneity, which allows for better standardization compared to natural lesions in human teeth.^36^ Previous studies have shown that the caries infiltration system, compared to other measures, generates the highest SBS values for brackets on demineralized enamel. This is attributed to its good penetration and effective micromechanical interlocking. Additionally, it was found that the bond strength values are not significantly different from those of healthy enamel.^21^ These observations were confirmed in our study.

The surfaces were etched with 35% phosphoric acid following the recommended bonding protocol before applying the primer and Transbond XT. Studies have demonstrated that phosphoric acid exerts erosive effects on infiltrated areas, although the erosion is more pronounced in natural enamel.^46^ Primer formulations with high concentrations of TEGDMA (triethylene glycol dimethacrylate) and HEMA (2-hydroxyethyl methacrylate) exhibit significant penetration capability. This likely facilitates a chemical bond between the resin infiltrant and the primer. Previous research indicates that the use of resin infiltrants does not negatively impact bond strength. Some studies even report increased SBSs with the use of Transbond XT in combination with phosphoric acid, attributed to enhanced micromechanical interlocking.^46^ There were no significant differences in SBS between sound and eroded enamel pretreated with RI, although the values tended to be higher on sound enamel. These findings align with previously published studies.^4,46^ Significant differences were observed within the home bleaching samples treated with 10% carbamide peroxide compared to the control group. However, there were no significant differences between the two bleaching methods used, despite descriptive differences in SBS values. This can be attributed to outliers with higher SBS values, which, after correction, did not allow for significance. Reynolds postulated that SBS values ranging from 5.9 to 7.8 MPa are sufficient for most clinical orthodontic applications.^39^ The optimum SBS value to avoid debonding is specified as 14 MPa.^35^ In this study, all groups exceeded the 5.9–7.8 MPa range postulated by Reynolds at both time points, indicating that bond strength remained sufficient for clinical orthodontic use throughout. Referred to the more demanding 14 MPa optimum, however, only the negative control and the resin-infiltrated group remained above this value after thermocycling, whereas both bleaching groups fell below it after aging.

The ARI index has proven useful in studies of orthodontic adhesives, as it allows for a quick and simple classification of the bonding failure mode.^3,21^ In our study, significant differences were found between the tested groups, but neither before nor after thermocycling within the groups. The ARI values were particularly high in the control group, indicating a high bonding ability between the composite and enamel. Conversely, the resin-infiltrated group (positive control group) and the in-office bleaching group exhibited slightly reduced values, consistent with the findings of Costenoble et al and Ekizer et al.^14,21^ The interlocking between composite, resin infiltration, and enamel might have been weaker in these groups. The home bleaching group showed the lowest ARI values, with bonding failure occurring mainly at the interface between composite and enamel. This suggests that the prolonged exposure time of home bleaching may have led to premature aging of the resin infiltration, resulting in the low ARI values. Cohesive failure in the enamel was partially observed. This suggests that the treatments conducted may have potentially negative effects on orthodontic bonding. However, the directions of the forces applied in clinical and experimental settings differ significantly. In the mechanical tests, force was applied along the occluso-gingival axis. In contrast, the removal of brackets in clinical practice is performed perpendicular to the tooth axis using a specialized instrument, known as a bracket removal plier. This approach aims to leave as much adhesive on the tooth as possible, improving control over the removal process and preserving the enamel.^17^ Previous studies have shown that the ARI index is not a direct reflection of bond strength.^37^ However, our results suggest a partial correlation between the ARI index and SBS in certain groups. Consequently, further studies are necessary to investigate the type of failures and their correlation with SBS in more detail.

Thermocycling was utilized to thermally stress the adhesive bond interface. Accelerated aging can alter the mechanical properties of resin materials and reduce hardness due to hydrolysis of the resin matrix.^38^ It is generally recommended to perform 3000 thermocycles between 5°C and 55°C to simulate the intraoral thermal cycling experienced during the typical orthodontic treatment period.^6^ However, in this study the thermocycling protocol was extended to 5000 cycles to more accurately replicate clinical conditions, as has been done in other studies.^6^ This decision was made to comprehensively evaluate the potential long-term effects of intensive thermal stresses and to better account for the variability in thermal stresses that different patients might experience. Although laboratory conditions cannot fully replicate the oral environment, they allow for controlled testing of specific variables, providing valuable data that can support predictions about clinical performance.

The SEM images are in accordance with the SBS and ARI results observed in control, resin infiltration, and in-office bleaching groups. The control and resin infiltration group exhibited a highly proximate contact between the adhesive, resin infiltrant, and enamel. In-office bleaching group with hydrogen peroxide exhibited some irregularities, but otherwise demonstrated a solid contact bond, thereby confirming the reliability of the orthodontic bonding in these groups. To date, only the study by Costenoble et al has examined the interfaces of resin infiltration on eroded enamel, as well as the interface of the adhesive.^14^ The results of our study are also consistent with those of previously published research. The supplementary examination of the bleaching impact in our study additionally demonstrated that bleaching can exert a detrimental influence on the hybrid layer between resin infiltration and the adhesive. The effects of in-office bleaching are minor and manifest as small voids or increased roughness, which can be attributed to the accelerated aging of the composite. However, the SEM image of the home bleaching group with the carbamide peroxide group demonstrates a notable loss of the hybrid layer between resin infiltration and adhesive, accompanied by gap formation. This finding is also consistent with the reduced SBS values observed in this group. The prolonged contact time of the bleaching gel results in notable resin infiltration weakness, which in turn reduces copolymerization with the adhesive. It is noteworthy that the existing SEM image shows significant irregularity, suggesting that in other samples, the hybrid layer may have been better formed.

Caution is needed when applying these findings to clinical practice, as the study was conducted in a controlled laboratory environment. Therefore, further clinical investigations under oral conditions are essential to validate the existing results. Additionally, studying the effects of various bleaching protocols on the shear bond strength of brackets on resin-infiltrated enamel would provide a more comprehensive understanding of the interaction between these treatments. Furthermore, sandblasting of the infiltrated and bleached enamel surfaces might improve shear bond strength values, especially for home bleaching, as has been shown for other substrates.^30,32^ Color changes should also be evaluated in future studies to identify potential correlations between adhesion strength and color alterations. It should also be considered that the SBS of buccal and lingual surfaces may inherently differ, which could serve as an inherent confounder.

## CONCLUSION

Treatment of white spot lesions with resin infiltration followed by subsequent bleaching negatively impacts bracket bonding. In our study, home bleaching with 10% carbamide peroxide significantly reduced shear bond strength, while in-office bleaching with 25% hydrogen peroxide showed no significant effect. Although no significant differences were observed between the bleaching methods overall, shear bond strengths were consistently lower with home bleaching. Additionally, thermocycling markedly reduced shear bond strengths across all treatment groups. Clinically, these values remained above recommended thresholds.

### Acknowledgments

#### Funding

This research did not receive any specific grant from funding agencies in the public, commercial, or not-for-profit sectors.

#### Conflict of interest

Michael Jochen Wicht reports a relationship with DMG Chemisch-Pharmazeutische Fabrik that includes speaking and lecture fees. All other authors declare that they have no known competing financial interests or personal relationships that could have appeared to influence the work reported in this paper.

#### Clinical relevance

Home bleaching (HB) should be avoided prior to orthodontic treatment and instead performed after to ensure optimal bracket adhesion and treatment success.

**Appendix 1:** Composition of solutions used in the study

	
Artificial saliva	22.1 mmol/L hydrogen carbonate, 16.1 mmol/L potassium, 14.1 mmol/L sodium hydrogen, 2.6 mmol/L phosphate, 0.8 mmol/L boric acid, 0.7 mmol/L calcium, 0.4 mmol/L thiocyanate and 0.2 mmol/L magnesium with pH ranging between 7.4 and 7.8
Demineralization solution	2.0 mmol/L calcium, 2.0 mmol/L phosphate, and 75 mmol/L acetate at pH 4.3
Remineralization solution	1.5 mmol/L calcium, 0.9 mmol/L phosphate, 150 mmol/L potassium chloride, and 20 mmol/L cacodylate buffer at pH 7.0

